# ARBitR: an overlap-aware genome assembly scaffolder for linked reads

**DOI:** 10.1093/bioinformatics/btaa975

**Published:** 2020-11-20

**Authors:** Markus Hiltunen, Martin Ryberg, Hanna Johannesson

**Affiliations:** Department of Organismal Biology, Uppsala University, 75236 Uppsala, Sweden; Department of Organismal Biology, Uppsala University, 75236 Uppsala, Sweden; Department of Organismal Biology, Uppsala University, 75236 Uppsala, Sweden

## Abstract

**Summary:**

Linked genomic sequencing reads contain information that can be used to join sequences together into scaffolds in draft genome assemblies. Existing software for this purpose performs the scaffolding by joining sequences with a gap between them, not considering potential overlaps of contigs. We developed ARBitR to create scaffolds where overlaps are taken into account and show that it can accurately recreate regions where draft assemblies are broken.

**Availability and implementation:**

ARBitR is written and implemented in Python3 for Unix-based operative systems. All source code is available at https://github.com/markhilt/ARBitR under the GNU General Public License v3.

**Supplementary information:**

Supplementary data are available at *Bioinformatics* online.

## 1 Introduction

Contiguity in genome assemblies is important for the ability to analyze e.g. structural rearrangements, gene order, synteny between divergent genomes, linkage between genetic variants, and repetitive genomic regions. Assembly contiguity can be improved by scaffolding: the use of long-range information to join assembled contigs into scaffolds. Such information can be found in linked genomic sequencing reads—short reads that are tagged with a region-specific barcode sequence during library preparation—allowing the investigator to determine which reads originated from regions in close proximity to each other (Bishara *et al.*, 2015; Zheng et al., 2016). Linked read technology was initially provided to a wide market by the 10X Genomics GemCode and Chromium systems (Eisenstein, 2015), and more recently, alternative methods such as Single Tube Long Fragment Read (stLFR) have been introduced (Wang et al., 2019).

Current linked-read scaffolding methods are based on 10X Chromium data and are composed of two steps: (i) finding linkage of original contigs using ARCS or ARKS (Yeo et al., 2018; Coombe *et al.*, 2018) and (ii) joining linked contigs into scaffolds using LINKS (Warren et al., 2015). ARCS relies on linked-read mappings while ARKS instead takes a kmer-based approach to avoid the mapping step, thus reducing computing time. After linkage has been determined, LINKS is called by the user to join the linked contigs with gaps in between. Such gaps can in some cases be resolved by filling them with read-derived sequence (Boetzer *et al.*, 2012). However, LINKS does not resolve cases where the original contigs overlap, instead it joins the contigs with a gap of size one. In genomes where repeat clusters are short, such overlaps may be quite frequent, and ignoring them leads to the risk of fragmenting genes and other features.

Here, we describe ARBitR: Assembly Refinement with Barcode-identity-tagged Reads. Compared to established pipelines, ARBitR has the advantages of performing the linkage-finding and scaffolding steps in succession in a single application, removing the need to install and run several software tools. Furthermore, during scaffolding, ARBitR considers overlaps between the involved contigs. While initially developed for 10X Chromium linked reads, ARBitR is also able to use stLFR reads, and can be adapted for any type of linked-read data.

## 2 Materials and methods

**Fig. 1.**
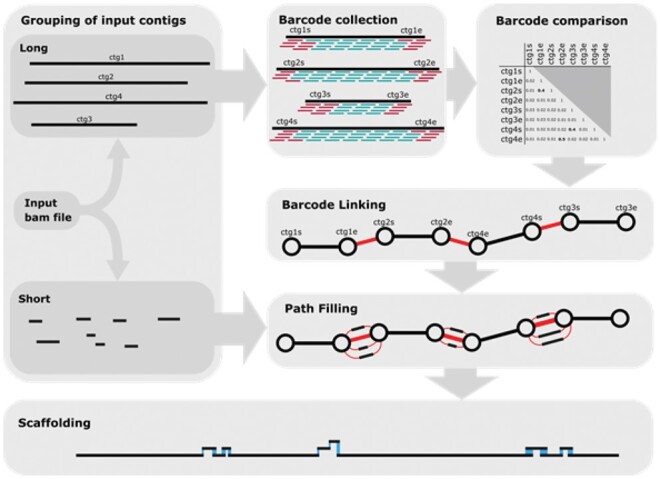
The ARBitR pipeline. To link contigs in the input assembly, ARBitR relies on barcode information of linked reads that have been mapped to the assembly. Short contigs are initially disregarded. From the starting (suffix s) and ending (suffix e) regions of the long contigs, barcodes are collected. For each region, the fraction of shared barcodes with every other region is computed, and regions that share a significantly high fraction are determined. Significant fractions are collected and represented in a graphical format, where nodes are input sequence start and end regions, and edges significant fractions of shared barcodes between these regions. Paths through the graph are determined, and at each step in the path, termed junction, ARBitR adds the short input contigs that share a high fraction of barcodes with the junction. Prior to finding overlaps between contigs, ARBitR trims away contig ends with low coverage (not shown in the figure). Finally, sequences are produced from the paths, by resolving each junction by overlap-layout-consensus. See Supplementary Methods for pipeline details

The ARBitR pipeline is described in detail in Figure 1 and Supplementary Methods.

To test the performance of ARBitR in relation to the ARCS/ARKS and LINKS pipelines, we utilized three datasets (Supplementary Table S1): (i) published PacBio, Nanopore and 10X Chromium linked reads of the fungus *Marasmius oreades*, (ii) publicly available PacBio and 10X Chromium reads of *Arabidopsis thaliana* and (iii) simulated PacBio and 10X Chromium data from a *Caenorhabditis elegans* reference genome. Long reads were assembled, and linked reads mapped to each assembly (Supplementary Methods).

Scaffolds were created from the three assemblies using ARBitR v0.2. For comparison, we used ARCS v1.1.1, both in default and in ARKS mode, in combination with LINKS, and for benchmarking we used Quast (Gurevich *et al.*, 2013), the Longranger WGS pipeline (https://www.10xgenomics.com) and the Long Terminal Repeat (LTR) Assembly Index statistic (LAI) (Ou *et al.*, 2018). Additionally, to investigate the breadth of ARBitR applicability, we tested its performance on a larger genome with different types of data. For this purpose, we utilized an assembly of the human cell line NA12878 that was based purely on 10X Chromium reads. Two linked-read datasets were used to scaffold this assembly: the same Chromium reads that the assembly was based on, and stLFR reads from the same cell line. Datasets and software parameters are described in detail in Supplementary Methods and Supplementary Table S1. Computations were performed on a Dell server on Ubuntu 18.04.3 using a maximum of 48 cores and with 503 Gb available memory.

## 3 Results

**Fig. 2.**
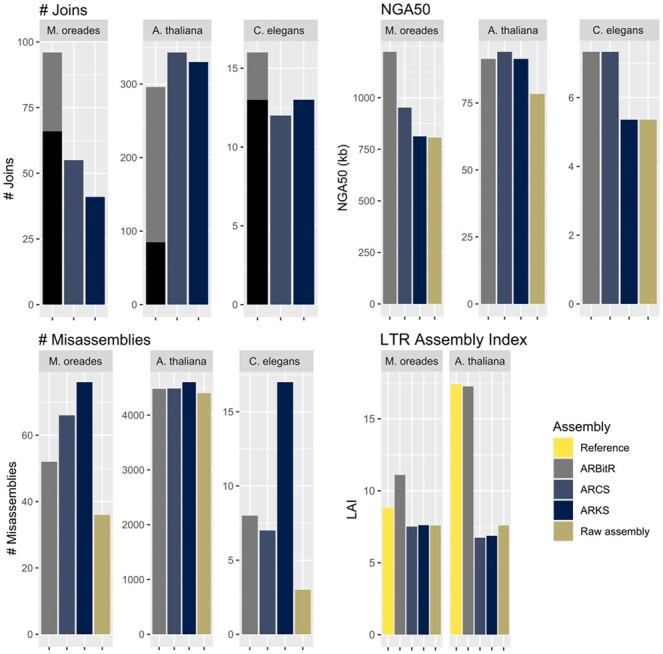
Scaffolding results. Top row: Number of joins (ARBitR aligned merges in black) and NGA50 for the three tested datasets. Bottom row: number of misassemblies and LTR assembly index (*C.elegans* had too few LTR elements to accurately calculate this statistic and was left out)

Scaffolding results of the three pipelines can be found in Figure 2 and Supplementary Table S2. ARBitR found a higher number of linked contigs in the *M.oreades* and *C.elegans* datasets than the other pipelines, and overlaps were found between the majority of the linked contigs in these datasets (# Joins in Fig. 2), leading to high NGA50 values. ARBitR scaffolds from most datasets contain the fewest misassemblies, base mismatches, indels and structural variants when comparing to reference assemblies. The highest LAI scores were found in ARBitR-scaffolded genomes, reflecting the advantage of overlap-aware scaffolding for assembling LTR elements. We noticed instances where genomic features appear fragmented or duplicated in LINKS scaffolds while being more complete in overlap merges performed by ARBitR (Supplementary Fig. S1). Compared to ARCS, ARBitR was faster, but sometimes at the cost of higher memory consumption (Supplementary Table S3). On the human data, ARBitR was able to improve the contiguity using both stLFR and 10X Chromium reads (Supplementary Table S4).

## 4 Conclusion

We present the new method ARBitR to apply linked-read information for scaffolding of draft genome assemblies. A key feature of the ARBitR pipeline is the consideration of overlaps between ends of linked contigs, which we found can decrease the number of erroneous structural variants, indels and mismatches in resulting scaffolds and improve assembly of transposable elements. Reducing the number of gaps this way diminishes the need to run a gap-filling algorithm after scaffolding, although investigators can still choose to do so in order to resolve remaining gaps. We expect ARBitR to have broad applicability in genome assembly projects that utilize linked reads, particularly in cases where repeat clusters are relatively short.

## Funding

This work was supported by the European Research Council (ERC) [ERC-2014-CoG] (project 648143, SpoKiGen) and the Swedish Research Council to H.J.

*Conflict of Interest*: none declared.

## Data Availability

The data underlying this article are available in the NCBI Sequence Read Archive under the accession numbers PRJNA525964, ERR3415826 and ERR2851508. URLs for the accessions, respectively: https://www.ncbi.nlm.nih.gov/bioproject/PRJNA525964/, https://www.ncbi.nlm.nih.gov/sra/?term=ERR3415826, https://www.ncbi.nlm.nih.gov/sra/?term=ERR2851508.

## Supplementary Material

btaa975_Supplementary_Data
