# Supplementary material for: ARBitR: an overlap-aware genome assembly scaffolder for linked reads
Source: Bioinformatics. 2020 Nov 20;37(15):2203–5. doi: 10.1093/bioinformatics/btaa975 (PMC8352505; doi:10.1093/bioinformatics/btaa975)
Supplement: btaa975_Supplementary_Data [file btaa975_supplementary_data.docx]

**Supplementary information**

**Supplementary methods**

*ARBitR algorithm details*

The ARBitR pipeline (Figure 1) initiates by sorting input contigs into small and large (threshold defined by -m). To determine the overarching structure of the scaffolds to be produced, ARBitR uses only the large contigs, as short contigs are often repeat rich and may obstruct efficient scaffolding because of unreliable read mapping. There are then six major steps of the ARBitR pipeline.

In the first step (Barcode Collection), the barcodes from the linked reads are collected from the start and end regions of the large contigs (region size defined by -s). Only reads with sufficient mapping quality are used (determined by -q), and only barcodes occurring in several reads are used (minimum number defined by -b). To collect barcodes, ARBitR utilizes the Pysam module (https://github.com/pysam-developers/pysam) which acts as a wrapper for samtools (Li et al., 2009).

In the second step (Barcode Comparison), the barcodes collected from every start and end region are compared to the barcodes in every other region. The fraction of shared barcodes is then computed by dividing the number of common barcodes in every pair of regions by the total number of barcodes in both regions in the same pair, counting each barcode only once. Results are stored in a NumPy array in memory and are written to disk (S. van der Walt *et al.*, 2011).

During the third step (Barcode Linking), links are computed from the fractions of shared barcodes. For each region, an outlier test is used to find linked regions. The upper bound *U* is calculated as

$$U=Q3+f*IQR$$

…where *Q3* is the upper quartile to the sample of fractions, *f* is a factor that can be changed by the user (-f) and *IQR* is the interquartile range. Links are formed between regions where their fraction of shared barcodes exceeds *U.* The additional Python modules SciPy and Pandas are utilized for these calculations (The pandas development team, 2020; Virtanen *et al.*, 2020). After finding the linkage between regions, ARBitR summarizes the results in a graphical data structure we refer to as a link graph. In the graph, nodes correspond to contig start and end regions, and edges to the newly found links between the regions. Paths through the graph are determined, where paths are terminated at nodes where the next step in the path cannot be unambiguously determined. The paths correspond to the major structure of the scaffolds that will be output by ARBitR. Each step in the path is termed a junction, and the barcodes from the source nodes in the junction are kept in memory. A junction has a start, a target, zero to many connections, and barcodes.

At the fourth step (Path Filling), ARBitR attempts to anchor the short contigs into the linkgraph. This is done by collecting the barcodes from the short contigs similarly as before (now instead with -Q as a mapping quality cutoff), and incorporating them into the junction connections if a high fraction of shared barcodes with the junction is found.

In the fifth step (Trimming), the ends of each contig in each path is trimmed for regions where coverage drops below a number that is determined by -c. The trimming is done to facilitate finding overlaps, as the sequence quality near the ends of contigs often deteriorates, leading to low read coverage after mapping. Only regions where there are potential overlaps are trimmed (e.g., if a path starts with contig1 in forward orientation, only the 3’ end of contig1 is trimmed while the 5’ end is kept intact).

At the sixth step (Scaffolding), the paths are joined into continuous sequence. At each junction, the involved contigs are aligned to each other using the module Mappy (Li, 2018), and an overlap graph is calculated. Paths between the junction start and end nodes are determined. If there is more than one, the shortest one that uses the highest number of contigs is used. If there are no complete paths between the start and target, as many contigs as possible are included. ARBitR does not estimate the gap distance, instead inserting a 100 base pair gap where the path breaks; the standard for gaps of unknown length. By incorporating the overlapping step, ARBitR has the potential to directly resolve the full sequence at a scaffold junction without the need for post-processing analyses such as gap filling.

*Scaffolding procedure of benchmarking datasets*

For the *Caenorhabditis elegans* dataset, we used PBSIM and LRSIM to simulate PacBio and 10X Chromium linked reads, respectively (Asai *et al.*, 2012; Luo *et al.*, 2017). For each of the three datasets, long reads were assembled using Canu (Koren *et al.*, 2017). A high-confidence reference genome of *Marasmius oreades*, that could be used to quality control our raw genome assembly, has yet to be published, and hence we subjected our raw assembly to an initial quality checkup using Tigmint prior to scaffolding (Jackman *et al.*, 2018). Tigmint uses 10X Chromium linked reads to spot regions in draft assemblies where the barcode coverage drops below a certain threshold, as such regions potentially have been wrongly assembled. We used tigmint-molecule to spot such regions (-s 10000) and custom scripts to break the assembly where such regions were found (available at <https://github.com/markhilt/genome_analysis_tools>). After draft assemblies were obtained for the three datasets, the linked reads were mapped using either BWA mem or EMA (Li *et al.*, 2009; Shajii *et al.*, 2018). Scaffolding was performed using ARBitR v0.2, ARCS v1.1.1 and LINKS v1.8.6 (Warren *et al.*, 2015; Yeo *et al.*, 2017; Coombe *et al.*, 2018). Computing resource consumption statistics were gathered using the time program in bash.

Parameters for ARBitR were chosen as follows: -s, controlling the size of the regions near contig ends where barcodes are collected, should be high enough to find good seeding regions, but not so high that a potential signal is diluted, and so is dependent on the size of input DNA for linked-read sequencing, in addition to the average length of repeat clusters in the genome under investigation. For *M. oreades*, both input molecule size and repeat regions were shorter than in the other genomes, leading us to choose a low -s in this case, higher in the case of *A. thaliana*, and even higher in *C. elegans* because the higher molecule length allowed to (simulated to 60 kb). Similarly, -m, the threshold for what is considered short and long contigs, is also dependent on repeat region lengths and molecule sizes, thus we chose it with the same reasoning as for -s. The mapq value when anchoring short contigs (-Q) depends on the difficulty in finding seeding regions in the short contigs, i.e. how similar the short contigs are, and were selected accordingly. The stringency for determining if two contigs are linked or not can be specified by -f, -r and -b, where noisier datasets, such as the *A. thaliana* dataset, require higher stringency. Thus, we selected higher values for these parameters for the *A. thaliana* dataset than the other ones. In the trimming step (see above), the minimum coverage required to stop trimming a contig end is determined by -c. We thus chose -c depending on the coverage of the linked-read datasets.

*Longranger variant calling*

As an additional measurement of genome concordance, we characterized variation between each assembly and the 10X Chromium reads, with the assumption that better assemblies have fewer called variants. For this purpose, we applied the Longranger WGS pipeline to all assemblies, for *M. oreades* and *A. thaliana* we used Longranger v2.2.2 and for *C. elegans* v2.1.4, as the newer version was incompatible with simulated reads. We quantified the number of calls with a PASS quality flag for large-scale structural variations, mid-scale deletions and single-nucleotide variants in addition to short indels and summarized in Table S2.

*Long Terminal Repeat analysis*

As genome assemblies are known to break in repetitive regions, and Long Terminal Repeat (LTR) transposable elements are the most common repeats in plant and fungal genomes (Ou and Jiang, 2018; Castanera *et al.*, 2016), we quantified the extent to which LTRs were assembled in the *M. oreades* and *A. thaliana* genomes (the low amount of LTR elements in the *C. elegans* genome made this analysis impossible in this case). The analysis was performed as recommended by the authors of LTR_retriever (Ou and Jiang, 2018): first find candidates of LTR elements in the genome using LTRharvest (-minlenltr 100 -maxlenltr 7000 -mintsd 4 -maxtsd 6 -motif TGCA -motifmis 1 -similar 85 -vic 10 -seed 20 -seqids yes) and LTR_finder (Ellinghaus *et al.*, 2008; Xu and Wang, 2007), then use LTR_retriever to quantify how common these elements are in the genome and calculate the LTR Assembly Index.

*Scaffolding of a human genome*

To test the performance of ARBitR on a large genome not based on long sequencing reads, we obtained the genome of the human cell line NA12878 sequenced and assembled by 10X Genomics from <https://www.10xgenomics.com/resources/datasets/>. We used two sets of linked reads to scaffold this genome with ARBitR: the same 10X Chromium reads that the assembly was based on and stLFR reads from the same cell line (Wang *et al.*  2019). The stLFR reads were first converted into a format supported by ARBitR using the script convert_fastq.py which is distributed with ARBitR. 10X Chromium reads were pre-processed with Longranger basic. Both read sets were mapped using BWA mem and ARBitR v0.2 was used for scaffolding. In these cases, the larger molecule lengths allowed for higher -m and -s, and we increased the stringency for anchoring small contigs by boosting -B, -Q and -F.

**Supplementary tables**

| Table S1. Datasets used for benchmarking ARBitR, ARCS and ARKS. | | | | | | | |
| --- | --- | --- | --- | --- | --- | --- | --- |
| Species | Long read data source | 10X Chromium data source | Read mapper | ARBitR parameters | ARCS parameters | ARKS parameters | Data reference |
| *Marasmius oreades* | SRA: PRJNA525964 | SRA: PRJNA525964 | EMA | -s25000 -Q15 -f10 -r0 -b5 -c5 | -e25000 | -D -t40 | (Hiltunen *et al.*, 2019) |
| *Arabidopsis thaliana* | SRA: ERR3415826 | ENA: ERR2851508 | BWA mem | -s35000 -m50000 -Q15 -r0.03 -f39 | default | -D -t40 | (Sun *et al.*, 2019, Jiao & Scheeberger 2020) |
| *Caenorhabditis elegans* | Simulated (PBSIM) | Simulated (LRSIM) | EMA | -m50000 -b4 -c0 | -c8 | -D -t40 | (Thompson *et al.*, 2015) |

| Table S2. Scaffolding performance of ARBitR, ARCS and ARKS. Bold font indicates values closest to the references for each parameter. | | | | | | | | | | | | | | |
| --- | --- | --- | --- | --- | --- | --- | --- | --- | --- | --- | --- | --- | --- | --- |
| Species | Assembly | # Scaffolds | # Joins (overlaps+gaps) | N50 (bp) | L50 | Longest scaffold (bp) | NGA50 (bp) | # mis-assemblies | Mismatches/100kb | Indels/100kb | Longranger SVs | Longranger mid-scale deletions | Longranger SNVs & short indels | LAI^a^ |
| *M. oreades* | Maror1^b^ | 39 | NA | 3564693 | 6 | 5044703 | NA | NA | NA | NA | 0 | 31 | 6412 | 8.82 |
|  | Raw assembly | 163 | NA | 733082 | 18 | 2593343 | 807306 | **36** | 17.17 | 71.77 | **0** | 111 | 29011 | 7.59 |
|  | ARBitR | **69** | **96 (66+30)** | **2571248** | **7** | **4642196** | **1222856** | 52 | **10.4** | **58.39** | **0** | 111 | **28702** | 11.10 |
|  | ARCS | 108 | 55 (0+55) | 1501983 | 9 | 4619992 | 952276 | 66 | 17.50 | 71.84 | **0** | 123 | 28915 | 7.51 |
|  | ARKS | 122 | 41 (0+41) | 1163884 | 12 | 2953429 | 812704 | 76 | 17.20 | 71.83 | **0** | **108** | 28961 | **7.61** |
| *A. thaliana* | TAIR10^b^ | 7 | NA | 23459830 | 3 | 30427671 | NA | NA | NA | NA | 2 | 849 | 877989 | 17.40 |
|  | Raw assembly | 771 | NA | 454218 | 79 | 2359306 | 78369 | **4401** | 905.48 | 298.23 | 28 | 1243 | 1089317 | 7.59 |
|  | ARBitR | 476 | 296 (85+211) | 4422061 | 10 | 10670333 | 91453 | 4480 | **900.07** | **294.96** | 4 | 1555 | **1083449** | **17.25** |
|  | ARCS | **428** | **343 (0+343)** | 5267787 | 8 | 12501495 | **94086** | 4486 | 902.16 | 298.38 | **0** | 1558 | 1084159 | 6.74 |
|  | ARKS | 441 | 330 (0+330) | **6950158** | **7** | **12820601** | 91453 | 4602 | 904.76 | 298.32 | 13 | **1180** | 1085071 | 6.87 |
| *C. elegans* | PRJNA275000^b^ | 7 | NA | 17183857 | 3 | 20182852 | NA | NA | NA | NA | 6 | 0 | 6494 | NA |
|  | Raw assembly | 66 | NA | 5629213 | 7 | 12209780 | 5358954 | **3** | **1.74** | 3.30 | 82 | 2 | 8479 | NA |
|  | ARBitR | **50** | **16 (13+3)** | **10208130** | **4** | **20105543** | **7333048** | 8 | 1.94 | **3.10** | **42** | **0** | 8528 | NA |
|  | ARCS | 54 | 12 (0+12) | 8544338 | 5 | 13042304 | **7333048** | 7 | 1.97 | 3.29 | 60 | 2 | **8423** | NA |
|  | ARKS | 53 | 13 (0+13) | 9420178 | **4** | 21146662 | 5358954 | 17 | 1.74 | 3.31 | 84 | 2 | 8458 | NA |
| ^a^Long terminal repeat element Assembly Index | | | | | | | | | | | | | | |
| ^b^Reference genome | | | | | | | | | | | | | | |

| Table S3. Computing resource consumption of ARBitR, ARCS and ARKS. Total usage includes read mapping, sorting and indexing for ARBitR and ARCS (see Supplementary Table S1 for mapping details). Time is given in the format dd:hh:mm:ss. Lowest consumption in bold. | | | | | |
| --- | --- | --- | --- | --- | --- |
| Species | Scaffolder | User time (scaffolding) | User time (total) | Peak memory usage (scaffolding; Mb) | Peak memory usage total; Mb) |
| *M. oreades* | ARBitR | **00:00:01:24** | 00:23:52:36 | **374** | 45310 |
|  | ARCS | 00:00:12:29 | 01:00:21:29 | 544 | 45302 |
|  | ARKS | 00:07:53:14 | **00:07:53:14** | 668 | **668** |
| *A. thaliana* | ARBitR | **00:00:09:10** | 05:23:14:25 | 1367 | 14878 |
|  | ARCS | 00:00:40:42 | 06:00:24:16 | **1274** | 15089 |
|  | ARKS | 01:04:29:43 | **01:04:29:43** | 2217 | **2217** |
| *C. elegans* | ARBitR | **00:00:01:00** | 00:09:15:17 | 425 | 45406 |
|  | ARCS | 00:00:14:18 | 00:10:12:38 | **24** | 45406 |
|  | ARKS | 00:12:27:19 | **00:12:27:19** | 101 | **101** |

| Table S4. Scaffolding results of human cell line NA12878. Resource consumption includes mapping, sorting and indexing of linked reads. | | | | | | | | | | |
| --- | --- | --- | --- | --- | --- | --- | --- | --- | --- | --- |
| Assembly specifics | ARBitR parameters | # Scaffolds | # Joins (merges+gaps) | N50 (bp) | L50 | Longest scaffold (bp) | NGA50 | # mis-assemblies | User time (dd:hh:mm:ss) | Peak memory usage (Mb) |
| Raw assembly (Supernova) | NA | 15862 | NA | 43592034 | 21 | 109562015 | 2096213 | 4248 | NA | NA |
| stLFR scaffolds | -m 75000 -s 30000 -B 5 -F 40 -Q 60 -n 45 | 15787 | 76 (16+60) | 50287949 | 18 | 130050501 | 2111147 | 4312 | 02:06:39:32 | 40277 |
| 10X Chromium scaffolds | -m 75000 -s 40000 -B 20 -F 70 -Q 60 -n 3 | 15785 | 81 (16+65) | 50297199 | 18 | 130050651 | 2111341 | 4304 | 27:07:28:42 | 29818 |

**Supplementary figures**


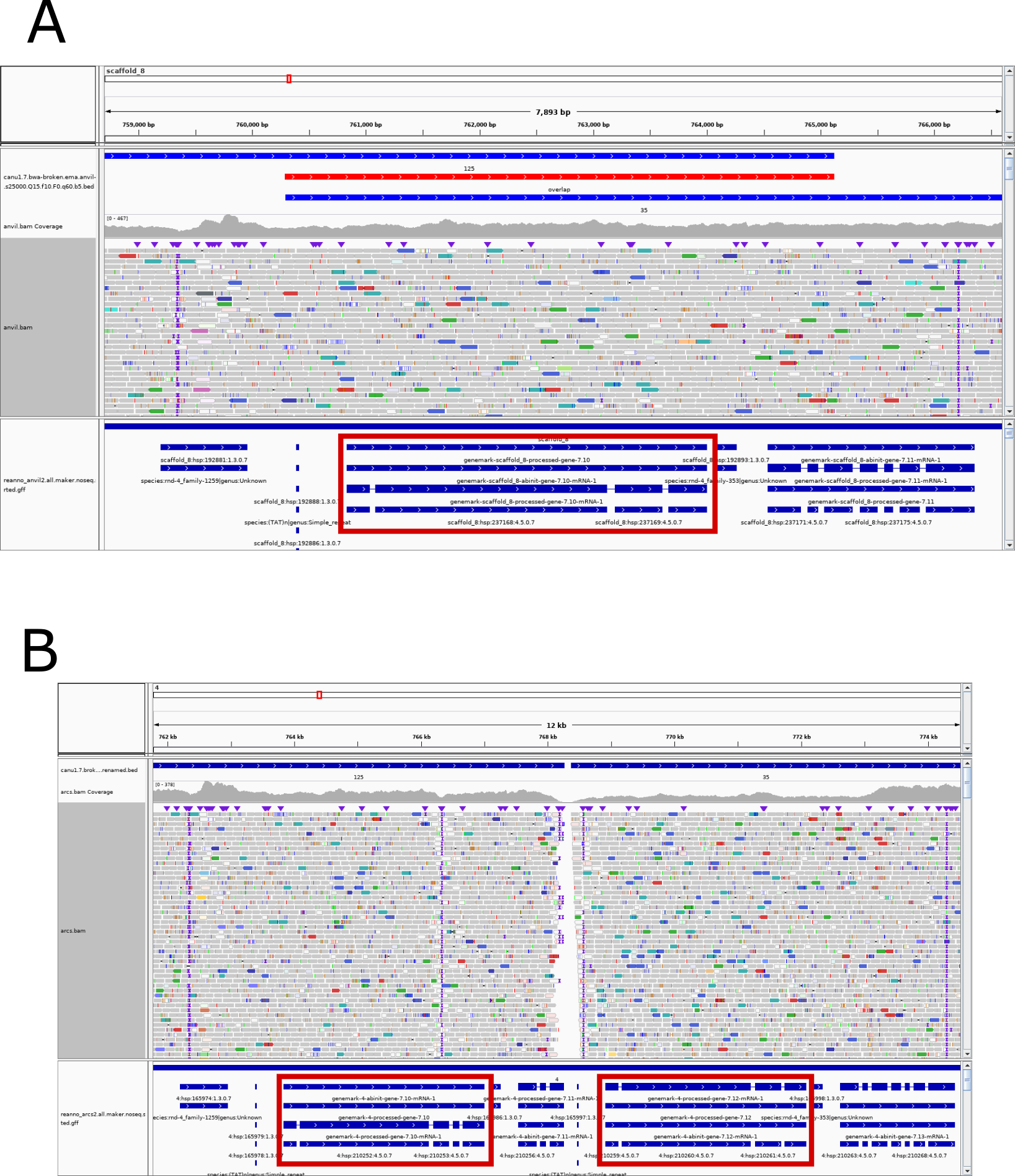


**Figure S2: Examples of scaffolding results.** IGV plots of (A) ARBitR and (B) ARCS & LINKS scaffolding results in a region where ARBitR performed an overlap merge in the *Marasmius oreades* dataset. Tracks from top to bottom: original scaffolds (overlap in red), mapped read coverage, read alignments, gene annotation. Highlighted: duplicated gene in the ARCS assembly. Note also the reduced read coverage in these regions in the ARCS assembly.

**Supplementary references**

Asai,K. *et al.* (2012) PBSIM: PacBio reads simulator—toward accurate genome assembly. *Bioinformatics*, **29**, 119–121.

Castanera,R. *et al.* (2016) Transposable Elements versus the Fungal Genome: Impact on Whole-Genome Architecture and Transcriptional Profiles. *PLOS Genetics*, **12**, e1006108.

Coombe,L. *et al.* (2018) ARKS: chromosome-scale scaffolding of human genome drafts with linked read kmers. *BMC Bioinformatics*, **19**, 234.

Ellinghaus,D. *et al.* (2008) LTRharvest, an efficient and flexible software for de novo detection of LTR retrotransposons. *BMC Bioinformatics*, **9**, 18.

Hiltunen,M. *et al.* (2019) Maintenance of High Genome Integrity over Vegetative Growth in the Fairy-Ring Mushroom Marasmius oreades. *Current Biology*, **29**, 2758-2765.e6.

Jackman,S.D. *et al.* (2018) Tigmint: Correcting Assembly Errors Using Linked Reads From Large Molecules. *BMC Bioinformatics*, **19**, 393.

Jiao,W. and Schneeberger,K. (2020) Chromosome-level assemblies of multiple *Arabidopsis* genomes reveal hotspots of rearrangements with altered evolutionary dynamics. *Nature Communications* **11,**989.

Koren,S. *et al.* (2017) Canu: scalable and accurate long-read assembly via adaptive *k* -mer weighting and repeat separation. *Genome Research*, **27**, 722–736.

Li,H. (2018) Minimap2: pairwise alignment for nucleotide sequences. *Bioinformatics*, **34**, 3094–3100.

Li,H. *et al.* (2009) The Sequence Alignment/Map format and SAMtools. *Bioinformatics*, **25**, 2078–2079.

Luo,R. *et al.* (2017) LRSim: A Linked-Reads Simulator Generating Insights for Better Genome Partitioning. *Computational and Structural Biotechnology Journal*, **15**, 478–484.

Ou,S. and Jiang,N. (2018) LTR_retriever: A Highly Accurate and Sensitive Program for Identification of Long Terminal Repeat Retrotransposons. *Plant Physiol.*, **176**, 1410.

S. van der Walt *et al.* (2011) The NumPy Array: A Structure for Efficient Numerical Computation. *Computing in Science & Engineering*, **13**, 22–30.

Shajii,A. *et al.* (2018) Statistical Binning for Barcoded Reads Improves Downstream Analyses. *Cell Systems*, **7**, 219-226.e5.

Sun,H. *et al.* (2019) Linked-read sequencing of gametes allows efficient genome-wide analysis of meiotic recombination. *Nature Communications*, **10**, 4310.

The pandas development team (2020) pandas-dev/pandas: Pandas Zenodo.

Thompson,O.A. *et al.* (2015) Remarkably Divergent Regions Punctuate the Genome Assembly of the Caenorhabditis elegans Hawaiian Strain CB4856. *Genetics*, **200**, 975–989.

Virtanen,P. *et al.* (2020) SciPy 1.0: Fundamental Algorithms for Scientific Computing in Python. *Nature Methods*, **17**, 261–272.

Wang,O. et al. (2019) Efficient and unique cobarcoding of second-generation sequencing reads from long DNA molecules enabling cost-effective and accurate sequencing, haplotyping, and de novo assembly. *Genome Res*, **29**, 798–808.

Warren,R.L. *et al.* (2015) LINKS: Scalable, alignment-free scaffolding of draft genomes with long reads. *GigaScience*, **4**, 35–35.

Xu,Z. and Wang,H. (2007) LTR_FINDER: an efficient tool for the prediction of full-length LTR retrotransposons. *Nucleic Acids Res*, **35**, W265–W268.

Yeo,S. *et al.* (2017) ARCS: scaffolding genome drafts with linked reads. *Bioinformatics*, **34**, 725–731.
